# Stress hyperglycemia indexes and early neurological deterioration in spontaneous intracerebral hemorrhage

**DOI:** 10.1007/s10072-025-08097-8

**Published:** 2025-03-19

**Authors:** Carmelo Tiberio Currò, Federica Ferrari, Giovanni Merlino, Stefan Moraru, Francesco Bax, Fedra Kuris, Lorenzo Nesi, Mariarosaria Valente, Elena Ballante, Nicola d’Altilia, Cristina Rascunà, Andrea Morotti, Federico Mazzacane, Anna Maria Cavallini

**Affiliations:** 1 Department of Brain and Behavioural Sciences, University of Pavia, Viale Golgi 19, Pavia, 27100 Italy; 2 Department of Cerebrovascular Disease/Stroke Unit, IRCCS Mondino Foundation, Via Mondino 2, Pavia, 27100 Italy; 3 Department of Head-Neck and Neuroscience, Stroke Unit, Udine University Hospital, Piazzale Santa Maria della Misericordia 15, Udine, 33100 Italy; 4 Department of Medical Area, University of Udine, Via Colugna 50, Udine, 33100 Italy; 5 Political and Social Sciences, University of Pavia, Corso Carlo Alberto 3, Pavia, 27100 Italy; 6 BioData Science Center, IRCCS Mondino Foundation, Via Mondino 2, Pavia, 27100 Italy; 7 Neurology Unit, Department of Clinical and Experimental Sciences, University of Brescia, Brescia, 25123 Italy

**Keywords:** Spontaneous intracerebral hemorrhage, Early neurological deterioration, Stress hyperglycemia, Glycemic gap, Stress hyperglycemia ratio, Glucose-glycated hemoglobin ratio

## Abstract

**Aim:**

To evaluate the relationship of early neurological deterioration (END) with admission glycemia (aG) and new stress hyperglycemia indexes in spontaneous intracerebral hemorrhage (ICH) patients.

**Methods:**

The present retrospective study included 171 ICH patients from two stroke centers. END was defined as an increase ≥ 4 points in National Institutes of Health Stroke Scale and/or a decrease ≥ 2 points in Glasgow Coma Scale within 72 hours from admission. The included stress hyperglycemia indexes were glycemic gap (GGAP), stress hyperglycemia ratio (SHR), and glucose-glycated hemoglobin ratio. GGAP was calculated as aG – 28,7*glycated hemoglobin + 46,7; SHR as aG / (28,7*glycated hemoglobin – 46,7); Glucose-glycated hemoglobin ratio as aG / glycated hemoglobin. We performed univariate and multivariate analyses for END. The receiver operating characteristic curves were built for END-related glycemic measures; area under curves (AUC) were calculated and compared. The optimized threshold values were calculated, and significant glycemic measures were dichotomized. Univariate and multivariate analyses were performed for the dichotomized measures.

**Results:**

END was present in 21 patients (12.3%) and was significantly associated with GGAP, SHR and glucose-glycated hemoglobin ratio, but not with aG. The AUC of the three stress hyperglycemia indexes did not differ significantly. The optimized cutoffs were 35.68 (sensitivity 0.47, specificity 0.81), 1.15 (sensitivity 0.62, specificity 0.68), and 26.67(sensitivity 0.43, specificity 0.80) for GGAP, SHR, and glucose-glycated hemoglobin ratio respectively. END was also associated with all stress hyperglycemia indexes expressed as categorical variables.

**Conclusion:**

GGAP, SHR, and glucose-glycated hemoglobin ratio were predictors of END in ICH patients.

**Supplementary Information:**

The online version contains supplementary material available at 10.1007/s10072-025-08097-8.

## Introduction

Spontaneous intracerebral hemorrhage (ICH) is a devastating disease that represents 10–20% of all acute cerebrovascular events [1]. Early neurological deterioration (END) is common, occurring in up to 40% of ICH patients [2–4]; a recent metanalysis of 32 studies, including 5014 patients, confirmed the high rate of END after ICH, showing a pooled prevalence of 23% with 95% confidence interval (CI) of 21–26% [4]. Accurate identification of END predictors might improve its prevention and offer an opportunity to improve outcome. END was mainly associated with admission neurological severity, hematoma volume, intraventricular hemorrhage, intraventricular extension, hematoma expansion, and spot sign [4]. There was not a clear association between glycemia and END, although INTERACT3 showed that long term prognosis could be influenced by a tight glycemic control as part of a care bundle [5]. Several studies investigated the association between ICH and stress hyperglycemia indexes [6–14]. These indexes could better describe stress hyperglycemia compared to glycemic measures alone because they also account for prestroke glycemic levels. Most studies on stress hyperglycemia indexes investigated only Asian cohorts [8, 10, 12, 13, 15, 16] and only Chu et al. focused on END [10]. Furthermore, Chu et al. studied exclusively stress hyperglycemia ratio (SHR) and did not consider the other stress hyperglycemia indexes [10]. The present analysis aimed to evaluate the relationship between END and admission blood glucose, glycemic gap (GGAP), SHR, and glucose-glycated hemoglobin ratio in a Caucasian cohort.

## Methods

### Study design and study population

**Fig. 1:**
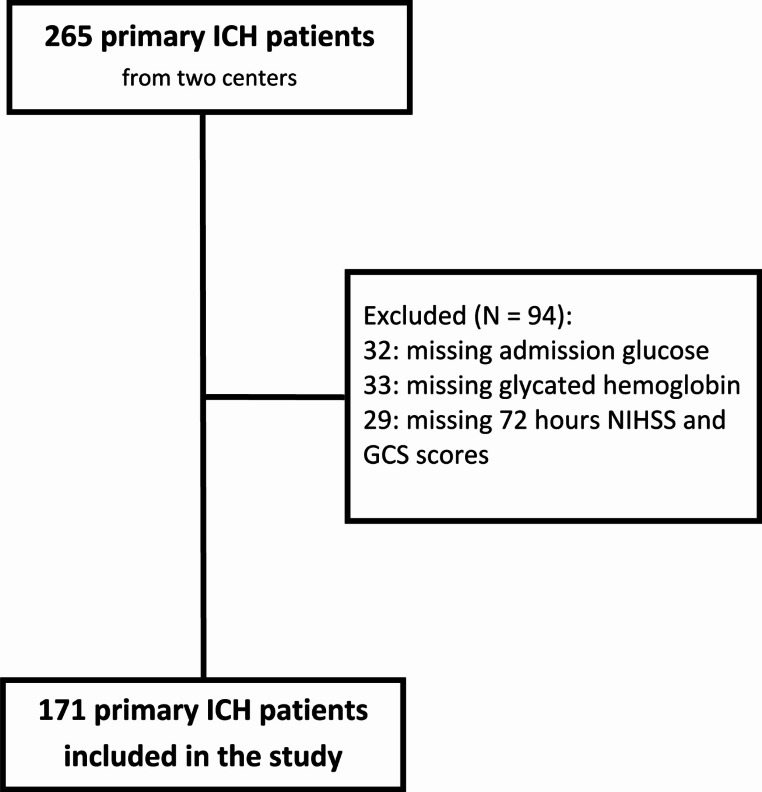
Included/excluded patients’ flow-chart

The present research was a retrospective study that involved two comprehensive stroke centers in Italy. Data were retrieved from the local ICH prospective registries. The research included ICH patients admitted to our centers between January 2017 and December 2023. We excluded patients without available data on admission glucose, glycated hemoglobin, and END. See Fig. 1.

### Data collection

#### Baseline characteristics and risk factors

The following baseline characteristics and risk factors were studied: age, gender, smoke, alcohol abuse, history of arterial hypertension, diabetes mellitus, atrial fibrillation, liver disease, chronic kidney disease, dementia, previous stroke or transient ischemic attack, pre-stroke disability on modified Rankin scale, use of antiplatelet drugs, anticoagulant drugs, and statins. Admission systolic and diastolic blood pressure were also evaluated.

#### Laboratory data and calculation of stress hyperglycemia indexes

Laboratory data included admission hemoglobin, count of leucocytes, neutrophils, monocytes, lymphocytes, platelets, and international normalized ratio (INR). Regarding glycemic measures, we evaluated admission glycemia in the emergency department, glycated hemoglobin, GGAP, SHR, and glucose-glycated hemoglobin ratio. The following formulae were used to calculate stress hyperglycemia indexes:

$$ \begin{array}{l}GGAP\, = \,admission\,glycemia\,-\,\\\left( {28.7*glycated\,hemoglobin} \right)\, + \,46.7\end{array} $$

$$ \begin{array}{l}SHR\, = \,admission\,glycemia\,/\\\,\left[ {\left( {28,7*glycated\,hemoglobin} \right)\,-\,46,7} \right]\end{array} $$

$$ \begin{array}{l}Glucose-glycated\,hemoglobin\,ratio\, = \,\\admission\,glycemia\,/\,glycated\,hemoglobin\end{array}$$

During the hospitalisation, the hypoglycemic therapy was administered according to Italian stroke guidelines.

#### Neuroradiological and neurofunctional features

We evaluated admission ICH volume, location, side, intraventricular hemorrhage, subarachnoid hemorrhage, and hematoma expansion. ICH volume was calculated using ABC/2 method. Hematoma expansion was defined as a volume increase ≥ 6 ml and/or a growth ≥ 33% on follow-up CT scan.

Admission neurological severity was assessed using the National Institutes of Health Stroke Scale (NIHSS) and Glasgow Coma Scale (GCS). END was defined as an increase ≥ 4 points in NIHSS and/or a decrease ≥ 2 points in GCS within 72 h from hospital admission [17].

ICH score was also calculated, combining neuroradiological features (ICH volume, IVH, ICH location) and clinical parameters (age, GCS).

### Statistical analysis

Continuous variables were expressed using median and interquartile range (IQR). Shapiro–Wilk test was applied to evaluate the normal distribution. Categorical variables were described as absolute frequencies and percentages. The study population was divided according to the occurrence of END. Mann–Whitney U or Student’s t test for independent samples were performed to evaluate the association between END and continuous variables as appropriate. The chi-square or Fisher’s exact test were used to study the relationship between END and categorical variables. Multivariate analyses were performed to assess the association between END and other variables. Two models of logistic regression were developed for each glycemic parameter that was significant in univariate analysis. The model 1 included all remaining variables that were significant in univariate analysis. The model 2 included the hematoma expansion and the model 1 variables. The backward elimination method (at *P*-value > 0.1) was used.

A receiver operating characteristic (ROC) was produced to evaluate the predictive utility of each stress hyperglycemia index for END. Further ROC curves were plotted for each multivariate model including and excluding the stress hyperglycemia indexes. The area under the curve (AUC) was calculated to measure the accuracy. Delong’s test was applied to compare the different curves. Youden’s J static was also performed in order to find the cut off values which maximised sensitivity and specificity of stress hyperglycemia indexes. Positive predictive value (PPV) and the negative predictive value (NPV) were calculated for every cut off value. GGAP, SHR, and glucose-glycated hemoglobin ratio were also dichotomized according to the results of Youden’s J static. The univariate and multivariate analyses of END were also performed for each stress hyperglycemia index expressed as categorical variable using the statistical tests mentioned above. Furthermore, crude and adjusted odds ratio (OR) were calculated for each stress hyperglycemia indexes. The adjustments were made for the variables included in final model 1 and in final model 2. A *P*-value ≤ 0.05 was considered significant. The statistical analysis was performed with R software version 4.3.2.

### Compliance with ethical standards

The study was conducted according to the World Medical Association Declaration of Helsinki. The research was approved by the institutional review board of each center. Every patient or a legal representative provided a written informed consent to data collection and publication for research purposes.

## Results

### Descriptive analyses

A total of 171 ICH patients were included in the present study. The study cohort selection is summarized in Fig. 1. The median age was 77.88 (68.05–83.06) and ninety-seven patients (56.7%) were male. One hundred fifty-three (89.5%) suffered by arterial hypertension, and forty-seven (27.5%) had diabetes mellitus. The median admission blood glucose was 126.00 (106.50-163.50) mg/dL, and the glycated hemoglobin was 5.80 (5.40–6.35) %. GGAP was 5.37(-13.29-30.72) mg/dL, SHR was 1.05 (0.90–1.26), and glucose-glycated hemoglobin ratio resulted 21.43 (18.44–26.27). The admission ICH volume amounted to 8.81 (3.42–25.82) mL. Intraventricular hemorrhage was present in fifty-two patients (30.4%) and subarachnoid hemorrhage in thirty patients (17.5%). The median ICH score amounted to 1 (0–2). Hematoma expansion was found in fifty-five cases (32.2%). END was present in twenty-one patients (12.3%). The present and remaining data were summarized in Table 1. There were no critical missing values, see table S1.

**Table 1:** Baseline characteristics, intracerebral hemorrhage features and early neurological deterioration (END)

		Early neurological deterioration		
	Overall 171	Yes 21	No 150	*P*
**Age** years, median (IQR)	77.88 (68.05–83.06)	78.97 (69.82–83.88)	77.71 (68.02–82.73)	0.39
**Sex** n (%)				0.78
Male	97 (56.7)	13 (61.9)	84 (56.0)	
Female	74 (43.3)	8 (38.1)	66 (44.0)	
**Arterial hypertension** n (%)				1
Yes	153 (89.5)	19 (90.5)	134 (89.3)	
No	18 (10.5)	2 (9.5)	16 (10.7)	
**Diabetes mellitus n (%)**				0.88
Yes	47 (27.5)	5 (23.8)	42 (28.0)	
No	124 (72.5)	16 (76.2)	108 (72.0)	
**Atrial fibrillation n (%)**				1
Yes	29 (17.0)	3 (14.3)	26 (17.3)	
No	142 (83.0)	18 (85.7)	124 (82.7)	
**Liver disease n (%)**				0.26
Yes	8 (4.7)	2 (9.5)	6 (4.0)	
No	163 (85.3)	19 (90.5)	144 (96.0)	
**Chronic kidney disease n (%)**				0.68
Yes	14 (8.2)	2 (9.5)	12 (8.0)	
No	157 (91.8)	19 (90.5)	138 (92.0)	
**Smoke** n (%)				0.31
Yes	44 (26.5)	8 (38.1)	36 (24.8)	
No	127 (73.5)	13 (61.9)	109 (75.2)	
**Alcohol** n (%)				0.41
Yes	4 (2.4)	1(5.0)	3 (2.1)	
No	167 (97.6)	19 (95.0)	142 (97.9)	
**Dementia** n (%)				0.48
Yes	22 (12.9)	4 (19.0)	18 (12.0)	
No	149 (87.1)	17 (81.0)	132 (88.0)	
**Previous stroke/TIA** n (%)				1
Yes	29 (17.0)	3 (14.3)	26 (17.3)	
No	142 (83.0)	18 (85.7)	124 (82.7)	
**Prestroke mRs**, median (IQR)	0 (0–1)	0 (0–1)	0 (0–1)	0.53
**Antiplatelet drugs** n (%)				0.76
Yes	58 (33.9)	6 (28.6)	52 (34.7)	
No	103 (66.1)	15 (71.4)	98 (65.3)	
**Anticoagulant drugs** n (%)				1
Yes	26 (15.2)	3 (14.3)	23 (15.3)	
No	145 (84.8)	18 (85.7)	127 (84.7)	
**Statins** n (%)				0.17
Yes	40 (23.4)	2 (9.5)	38 (25.3)	
No	131 (76.6)	19 (90.5)	112 (74.7)	
**Systolic pressure** mmHg, median (IQR)	160 (150–182)	160 (131–185)	160 (150-180.5)	0.26
**Diastolic pressure** mmHg, median (IQR)	90 (80–100)	80 (70–95)	90 (80–100)	0.02
**Creatinine** mg/dL*, median (IQR	0.84 (0.69–0.99)	0.86 (0.70–1.01)	0.84 (0.69–0.98)	0.5
**Hemoglobin** g/dL *, median (IQR)	13.50 (12.55–14.40)	12.9 (12.2–13.7)	13.6 (12.8–14.4)	0.21
**Leucocytes** 10^3^ cells/mm^3^*, median (IQR)	8.99 (7.22–10.96)	8.60 (6.80–13.5)	9.05 (7.30–10.80)	0.93
**Neutrophils** 10^3^ cells/mm^3^*, median (IQR)	6.49 (5.10–8.37)	6.22 (4.90-11.49)	6.55 (5.10–8.29)	0.81
**Monocytes** 10^3^ cells/mm^3^*, median (IQR)	0.60 (0.50–0.80)	0.50 (0.49–0.84)	0.60 (0.50–0.80)	0.81
**Lymphocytes** 10^3^ cells/mm^3^*, median (IQR)	1.30 (0.95–1.79)	1.19 (0.70–1.50)	1.38 (0.98–1.80)	0.17
**Platelets x 10**^**3**^ cells/mm^3^*, median (IQR)	202.0 (165.0-245.5)	211.00 (158.00-262.00)	199.50 (166.25-242.25)	0.74
**INR**, median (IQR)	1.03 (0.98–1.12)	1.07 (1.02–1.17)	1.02 (0.98–1.11)	0.11
**Admission glycemia** mg/dL*, median (IQR)	126 (107–163)	145 (106–197)	124.5 (107–159)	0.07
**Glycated hemoglobin** % *, median (IQR)	5.80 (5.40–6.35)	5.6 (5.2–6.2)	5.8 (5.5–6.5)	0.3
**Glycemic gap** mg/dL, median (IQR)	5.37 (-13.29-30.72)	28.59 (-2.28-71.50)	4.46 (-13.86-29.83)	0.03
**SHR**, median (IQR)	1.05 (0.90–1.26)	1.25(0.98–1.57)	1.03 (0.89–1.21)	0.03
**GGHR**, median (IQR)	21.43 (18.44–26.27)	25.28 (19.63–32.83)	21.27 (18.32–25.83)	0.05
**Admission GCS**, median (IQR)	15 (13–15)	14 (11–15)	15 (13–15)	0.2
**Admission NIHSS**, median (IQR)	9 (6–17)	15 (8–18)	9 (5–16)	0.06
**Admission ICH volume** mL, median (IQR)	8.81 (3.42–25.82)	13.90 (4.32–56.58)	8.75 (3.41–23.27)	0.15
**ICH location** n (%)				0.22
Lobar supratentorial	63 (36.8)	7 (33.3)	56(37.3)	
Deep supratentorial	99 (57.9)	11 (52.4)	88 (58.7)	
Cerebellar	5 (2.9)	2 (9.5)	3 (2.0)	
Brainstem	4 (2.3)	1 (4.8)	3 (2.0)	
**ICH side** n (%)				0.82
Right	81 (47.7)	11 (52.4)	70 (47.0)	
Left	90 (52.3)	10 (47.6)	79 (53.0)	
**Intraventricular hemorrhage** n (%)				0.28
Yes	52 (30.4)	9 (42.9)	43 (28.7)	
No	119 (69.6)	12 (57.1)	107 (71.3)	
**Subarachnoid hemorrhage** n (%)				0.62
Yes	30 (17.5)	5 (23.8)	25 (16.7)	
No	141 (82.5)	16 (76.2)	125 (83.3)	
**ICH score**, median (IQR)	1 (0–2)	1 (1–2)	1 (0–2)	0.05
**Hematoma expansion** n (%)				0.06
Yes	55 (32.2)	11 (52.4)	44 (29.3)	
No	116 (67.8)	10 (47.6)	106 (70.7)	
**Early neurologic deterioration** n (%)				---
Yes	21 (12.3)	21 (100)	0 (0.0)	
No	150 (87.7)	0 (0.0)	150 (100)	

P: *p*-value; IQR: interquartile range; TIA: transitory ischemic attack; mRs: modified Rankin scale; INR: international normalized ratio; SHR: stress hyperglycemia ratio; GGHR: glycemic glycated hemoglobin ratio; GCS: Glasgow Coma Scale; NIHSS: National Institute of Health Stroke Scale; ICH: intracerebral hemorrhage;

* normal value: Creatinine (0.5–1.2), Hemoglobin (12.0–17.0), Leucocytes (4.5-9.0 × 10^3^), Neutrophils (1.5-7.0 × 10^3^), Monocytes (100–850), Lymphocytes (1.5-3.0 × 10^3^), Platelets (150.0-350.0 × 10^3^), INR (0.8–1.2), Blood glucose (65–110), Glycated hemoglobin (4.0–6.0%)

### Univariate and multivariate analyses including stress hyperglycemia indexes as continuous variables

The GGAP, SHR, and glucose-glycated hemoglobin ratio were significantly higher in END patients compared to NO END patients. The crude OR for GGAP were 1.02 (95% CI: 1.01–1.03, *p*-value = 0.03). Regarding SHR and glucose-glycated hemoglobin ratio, the crude OR were equal to 8.58 (95% CI: 2.31–35.08, *p*-value = 0.03) and 1.10 (95% CI: 1.04–1.18, *p*-value = 0.05), respectively. A statistically significant difference was not found for the admission blood glucose and the glycated hemoglobin. Furthermore, END was significantly associated with lower diastolic blood pressure and higher ICH score values. The results of univariate analysis between END and NO END patients were reported in Table 1.

The multivariate regression models confirmed the association between END and stress hyperglycemia indexes (see Table 2). Regarding GGAP, the adjusted OR was 1.01 (95% CI: 1.005–1.027, *p*-value = 0.01) for the final model 1 and 1.02 (95% CI: 1.006–1.030, *p*-value = 0.004) for the final model 2. The adjusted OR for SHR were 7.12 (95% CI: 1.85–30.95, *p*-value = 0.01) and 9.94 (95% CI: 2.55–43.82, *p*-value = 0.001) according to the final model 1 and the final model 2. Regarding glucose-glycated hemoglobin ratio, the adjusted OR were 1.09 (95% CI: 1.03–1.17, *p*-value 0.01) for the final model 1 and 1.11 (95% CI: 1.04–1.19, *p*-value = 0.002) for the final model 2. Diastolic blood pressure and hematoma expansion also resulted significantly associated with END in the final model 1 and 2, respectively.

**Table 2:** Multivariate analyses of early neurological deterioration (END) including stress hyperglycemia indexes as continuous variables

Glycemic gap						
**Model 1**						
	*Initial Model*			*Final Model*		
	OR	95% CI	*p*-value	OR	95% CI	*p*-value
Diastolic pressure	0.97	0.933–1.002	0.07	0.96	0.931–0.998	0.05
ICH score	1.32	0.862–1.999	0.19	----	----	----
GGAP	1.01	1.004–1.026	0.02	1.01	1.005–1.027	0.01
**Model 2**						
	*Initial Model*			*Final Model*		
	OR	95% CI	*p*-value	OR	95% CI	*p*-value
Diastolic pressure	0.97	0.937–1.005	0.11	----	----	----
ICH score	1.22	0.775–1.880	0.38	----	----	----
GGAP	1.01	1.004–1.027	0.02	1.02	1.006–1.030	0.004
Hematoma expansion	2.21	0.781–6.380	0.13	3.08	1.159–8.455	0.03

Stress hyperglycemia ratio						
**Model 1**						
	*Initial Model*			*Final Model*		
	OR	95% CI	*p*-value	OR	95% CI	*p*-value
Diastolic pressure	0.96	0.933–1.001	0.07	0.97	0.931–0.999	0.05
ICH score	1.31	0.857–1.987	0.20	----	----	----
SHR	6.17	1.56-27.042	0.01	7.12	1.849–30.946	0.01
**Model 2**						
	*Initial Model*			*Final Model*		
	OR	95% CI	*p*-value	OR	95% CI	*p*-value
Diastolic pressure	0.97	0.936–1.006	0.12	----	----	----
ICH score	1.21	0.771–1.868	0.39	----	----	----
SHR	7.26	1.740-33.781	0.01	9.94	2.545–43.824	0.001
Hematoma expansion	2.19	0.770–6.330	0.14	3.04	1.141–8.354	0.03

Glucose-glycated hemoglobin ratio						
**Model 1**						
	*Initial Model*			*Final Model*		
	OR	95% CI	*p*-value	OR	95% CI	*p*-value
Diastolic pressure	0.97	0.933–1.001	0.07	0.97	0.932–0.998	0.05
ICH score	1.32	0.867–1.999	0.19	----	----	----
GGR	1.09	1.019–1.165	0.01	1.09	1.026–1.171	0.01
**Model 2**						
	*Initial Model*			*Final Model*		
	OR	95% CI	*p*-value	OR	95% CI	*p*-value
Diastolic pressure	0.97	0.936–1.006	0.11	----	----	----
ICH score	1.22	0.778–1.878	0.37	----	----	----
GGR	1.09	1.025–1.176	0.01	1.11	1.042–1.191	0.002
Hematoma expansion	2.21	0.782–6.369	0.13	3.07	1.16–8.418	0.02

OR: Odd Ratio; 95% CI: 95% confidence interval; ICH: intracerebral hemorrhage; GGAP: glycemic gap; SHR: stress hyperglycemia ratio; GGR: glucose-glycated hemoglobin ratio

### ROC analyses

**Fig. 2:**
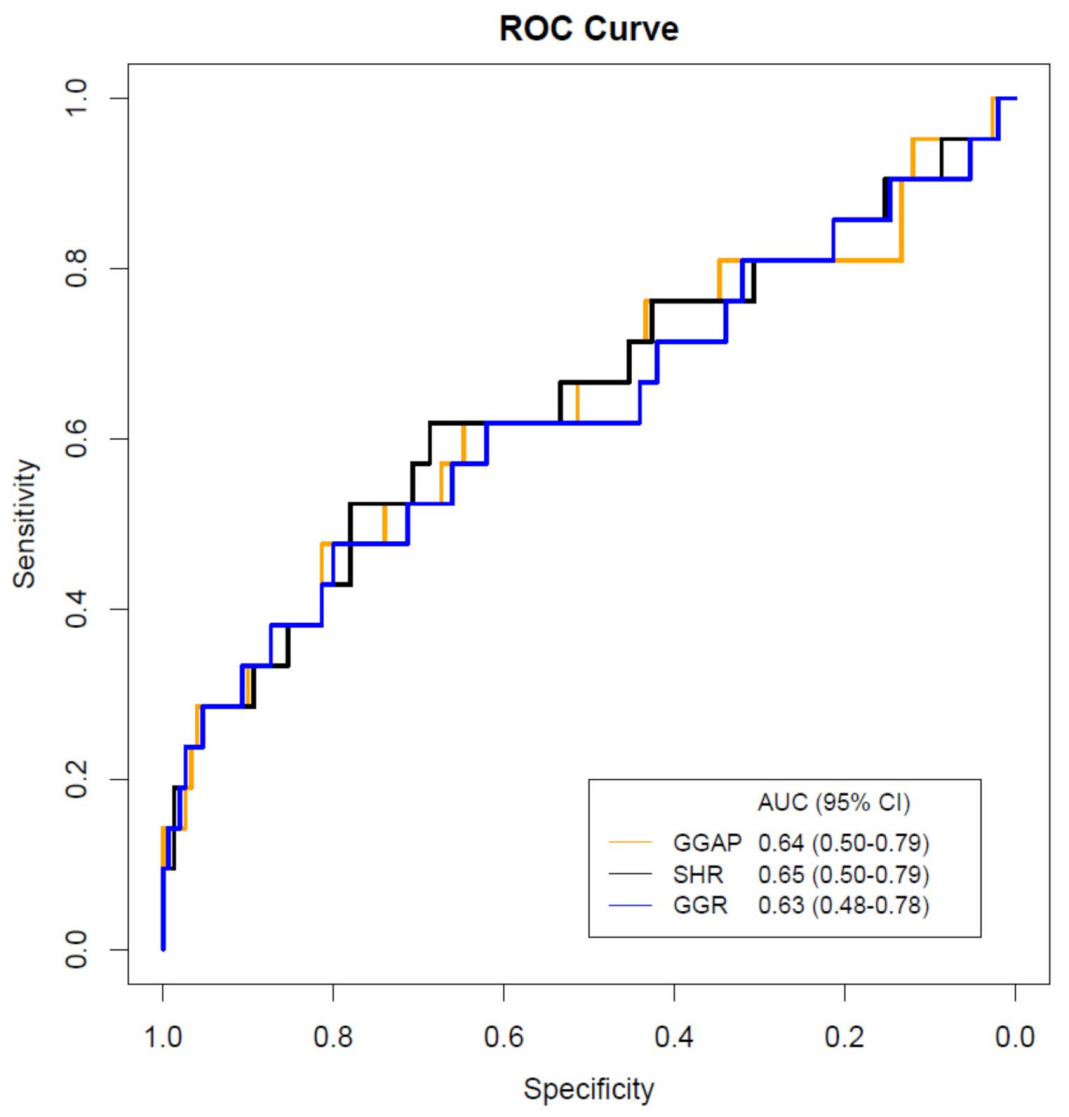
ROC curves of stress hyperglycemia indexes for predicting END AUC: area under the curve; CI: confidence interval; GGAP: glycemic gap; SHR: stress hyperglycemia ratio; GGR: glucose-glycated hemoglobin ratio

ROC curves of GGAP, SHR, and glucose-glycated hemoglobin ratio for predicting END were plotted in Fig. 2. SHR curve presented the largest AUC (0.65). The AUC of GGAP curve was 0.64, the value for glucose-glycated hemoglobin ratio was 0.63. Delong’s test did not show a significant difference when comparing the different curves.

**Fig. 3:**
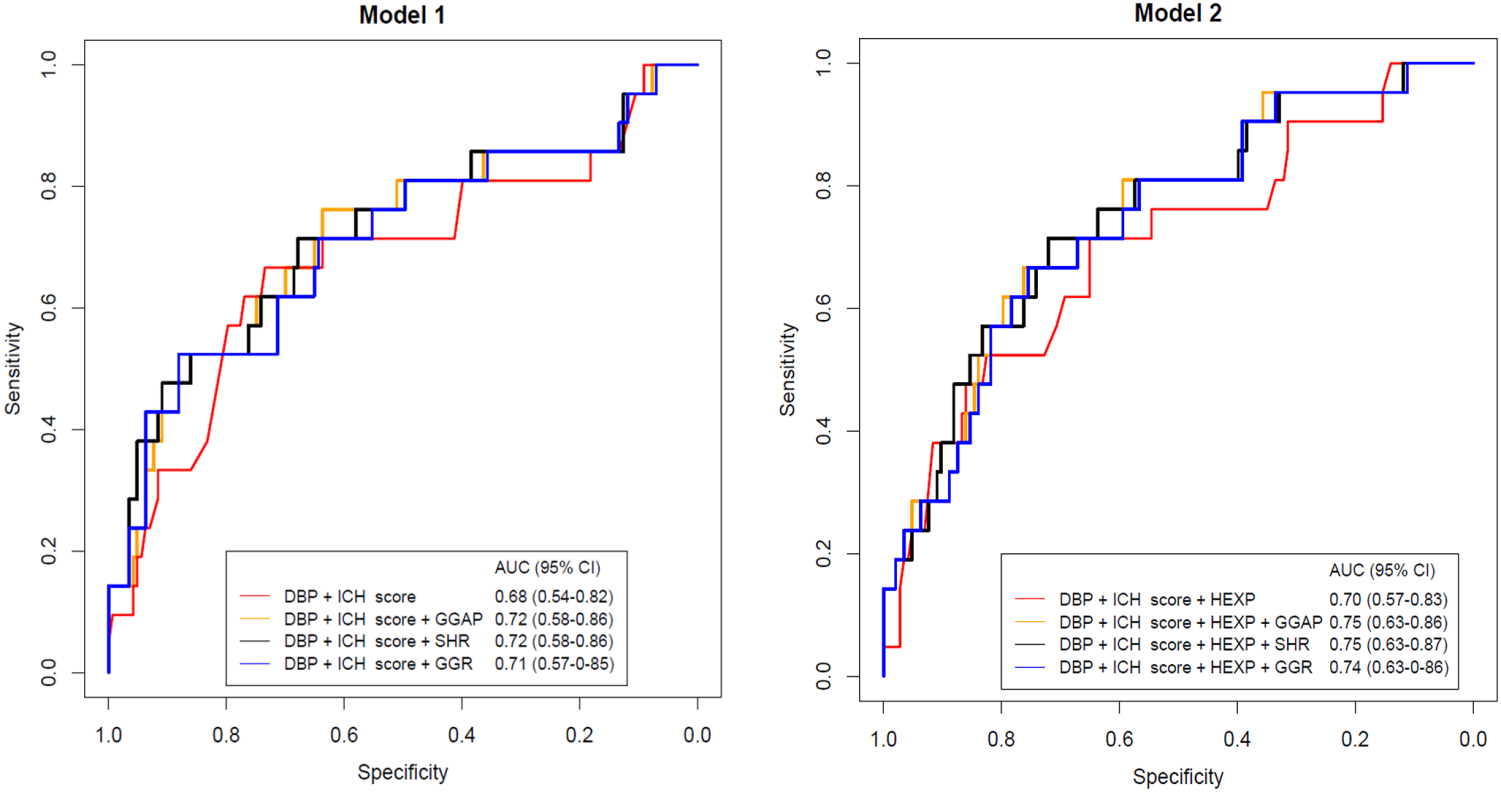
ROC curves for predicting END without and with stress hyperglycemia indexes in the Model 1 and Model 2 AUC: area under the curve; CI: confidence interval; DBP: diastolic blood pressure; ICH: intracerebral hemorrhage; HEXP: hematoma expansion; GGAP: glycemic gap; SHR: stress hyperglycemia ratio; GGR: glucose-glycated hemoglobin ratio

Model 1 ROC curves for predicting END with/without the stress hyperglycemia indexes were plotted in Fig. 3. The ROC curve without stress hyperglycaemia indexes had an AUC of 0.68. The AUC of Model 1 curves including the GGAP, SHR, and glucose-glycated hemoglobin ratio were 0.72, 0.72, and 0.71, respectively. Delong’s test did not show a significant difference when comparing the different curves of Model 1. Model 2 ROC curves with/without the stress hyperglycemia indexes were plotted in Fig. 3. Model 2 ROC curve without stress hyperglycaemia indexes had an AUC of 0.70. The AUC of Model 2 curves including the GGAP, SHR, and glucose-glycated hemoglobin ratio were 0.75, 0.75, and 0.74, respectively. Delong’s test did not show a significant difference when comparing the different curves of Model 2.

The optimized threshold for GGAP was 35.68 mg/dL with a sensitivity of 0.47 and a specificity of 0.81; the PPV was 0.26, and the NPV was 0.92. The cut-off value for SHR was 1.15 with a sensitivity of 0.62 and a specificity of 0.68; the PPV was 0.22, and the NPV was 0.93. The threshold for the glucose-glycated hemoglobin ratio was 26.67 with a sensitivity of 0.43, a specificity of 0.80, a PPV of 0.25, and a NPV of 0.92.

### Univariate and multivariate analyses including stress hyperglycemia indexes as categoric variables

Using the threshold values above reported, stress hyperglycemia indexes were transformed in dichotomic variables. A GGAP value ≥ 35.68 was present in thirty-eight patients; ten of whom experienced END. A SHR value ≥ 1.15 was found in sixty patients, and thirteen presented END. A glucose-glycated hemoglobin ratio ≥ 26.67 was present in forty patients, ten of whom experienced END.

Univariate and multivariate analyses were repeated to evaluate association between END and stress hyperglycemia indexes expressed as categoric variables (see Table S2). The analyses confirmed the GGAP, SHR, and glucose-glycated hemoglobin ratio as predictors of END. Regarding END and GGAP, the crude OR was 3.92 (95% CI: 1.48–10.34, *p*-value = 0.01), the adjusted OR was 3.49 (95% CI: 1.30–9.30, *p*-value = 0.01) in both final models. The crude OR for SHR were equal to 3.51 (95% CI: 1.37–9.53, *p*-value = 0.01), the adjusted OR was 3.32 (95% CI: 1.29–9.06, *p*-value = 0.01). The crude OR for glucose-glycated hemoglobin ratio corresponded to 3.60 (95% CI: 1.37–9.45, *p*-value = 0.01), the adjusted OR was 3.39 (95% CI: 1.27–9.02, *p*-value = 0.01) according to both final models. Furthermore, admission diastolic blood pressure was significantly associated with END in both final model 1 and 2.

## Discussion

The present analysis on ICH showed a positive association between different stress glycemic indexes and END, whereas no relationship was found between END and admission blood glucose. Furthermore, we compared accuracy of GGAP, SHR, and glucose-glycated hemoglobin ratio to predict END, and SHR presented a wider AUC but there was no significant difference with the other indexes.

Regarding the literature data about glycemia and early deterioration, several studies did not show a relevant association [4, 18–23]. In a meta-analysis of 2014 [24], blood glucose resulted as predictor of END, but the results were driven by the Franke et al. study, which evaluated only early death [25]. Considering only the studies focused properly on END in the aforementioned meta-analysis, these showed no association [18, 19]. Furthermore, glycemia was not considered as END predictor in a more recent meta-analysis of 2024 [4]. On the other hand, rather than glycemia per se, the stress hyperglycemia indexes are more informative because they evaluate both admission glycemia and usual glycemic levels calculated using glycated hemoglobin. A higher-than-usual value of glycemia could favour neurological deterioration and the present indexes offered a personalised measure of hyperglycemia. Each person could have a different glycemic tolerability, and these indexes are focused on this concept. The present research was not the first study to investigate relationship between END and stress hyperglycemia measures in ICH patients. Chu et al. found a significant association between END and SHR [10]. They analysed an Asiatic cohort of patients, whereas our study focused on a Caucasian cohort. Chu et al. defined END as a worsening of 3 point in GCS or the need of an early hemicraniectomy. We included patients with a decrease ≥ 2 points in GCS and/or an increase ≥ 4 points in NIHSS, which is the specific scale to evaluate neurologic severity in stroke patients. Furthermore, they considered a temporal window of 48 h, whereas we included deteriorations within 72 h. Another important point of our study was that we compared three different indexes of stress hyperglycemia. Although these differences between Chu et al. and our study, both analyses highlighted the importance of the stress hyperglycemia indexes.

The worsening due to stress hyperglycemia could be explained through several mechanisms. Stress hyperglycemia could favour neuroinflammation [26], oxidative stress [27], endothelial and mitochondrial dysfunction [28, 29], lactic acid accumulation [30], downregulation of aquaporin 4 [31], and the activity of matrix metalloproteinase 9 [32]. All these elements could lead to blood-brain barrier breakdown, neuronal apoptosis, increase of cytotoxic and vasogenic edema, and hematoma expansion [28, 30–33]. Furthermore, stress hyperglycemia could favour pneumonia and other infections [34, 35] that could play an important role in neurological worsening.

In the light of our results and literature data, the stress glycemic indexes could be instrumental in identifying a patient at risk of worsening. They are also inexpensive and easy-to-obtain, and it may be useful to include the glycated hemoglobin in laboratory tests required in the emergency department. Although GGAP, SHR, and GGR showed suboptimal AUC, sensitivity, and specificity in our study, these indexes could be used together with other END predictors [4] to better identify the high-risk patients. In fact, stress hyperglycemia treatment is a fundamental component of the INTERACT3 care bundle protocol [5] for prevention of neurological worsening in acute ICH, but embedded in a multimodal approach alongside other parameters. INTERACT3 indicated only one glycemic target for diabetic patients, but every diabetic patient has a different glycemic tolerance and insulin resistance. A different glycemic target could be adopted on the base of stress glycemic indexes. This concept could also be extended to non-diabetic patients. Furthermore, the neurologists often did not efficaciously treat hyperglycemia because they are too afraid of hypoglycemia. The stress hyperglycemia indexes could be useful in order to modulate the dosage of insulin and other antidiabetic therapy. INTERACT3 showed that bundle of care could ameliorate prognosis, but further advances are needed in ICH management.

The present study had several limitations. The retrospective design could represent a source of biases. END adjudicators were not blinded for glycemic status; however, END was based on NIHSS and GCS recorded in medical charts, and they were assigned before the glycemic indexes were calculated. Some missing data occurred. Patients without admission glycemia and glycated hemoglobin were excluded; however, this could have potentially led to the exclusion of patients who died prior to obtaining a blood sample for glycated hemoglobin. Furthermore, patients in more severe conditions could not be admitted to our departments, being otherwise admitted to Intensive Care Units. We analysed only a Caucasian cohort. A statistically significant association between stress hyperglycemia and END did not imply a direct causal relationship. Stress hyperglycemia could be not the cause of END, but an epiphenomenon of an infection or other processes involved in the neurological worsening. Glycemia had dynamic fluctuations in acute stroke patients, further studies should investigate the stress hyperglycemia with a continuous glucose monitoring [36] and evaluate glycemic temporal trends. The small number of patients limited our analysis and did not allow us to perform some subanalyses as an evaluation in only diabetic patients or in END patients without hematoma expansion. The stress hyperglycemia cutoffs should be adequately validated through other clinical studies, having here employed the same cohort both as “discovery” and “validation” one.

## Conclusions

The present study showed that SHR, GGAP, and glucose-glycated hemoglobin ratio were related to END in ICH patients. No stress hyperglycemia index resulted more accurate than others, and they represented inexpensive and easy-to-obtain biomarkers that could help the physician in the management of ICH.

## Electronic supplementary material

Below is the link to the electronic supplementary material.

Supplementary Material 1

Supplementary Material 2

## Data Availability

Research data supporting the present results are available from the corresponding author upon reasonable request.

## Abbreviations

END: Early neurological deterioration
GGAP: Glycemic gap
SHR: Stress hyperglycemia ratio
SICH: Spontaneous intracerebral hemorrhage
