# Supplementary material for: Stress hyperglycemia indexes and early neurological deterioration in spontaneous intracerebral hemorrhage
Source: Neurol Sci. 2025 Mar 19;46(7):3135–45. doi: 10.1007/s10072-025-08097-8 (PMC12152042; doi:10.1007/s10072-025-08097-8)
Supplement: Supplementary file 1 — Supplementary Material 1 [file 10072_2025_8097_MOESM1_ESM.docx]

**Table S1 Missing values for each variable**

|  |  |
| --- | --- |
|  | **Missing values**  **n (%)** |
| **Age** | 0 (0.0) |
| **Sex** | 0 (0.0) |
| **Arterial hypertension** | 0 (0.0) |
| **Diabetes mellitus** | 0 (0.0) |
| **Atrial fibrillation** | 0 (0.0) |
| **Liver disease** | 0 (0.0) |
| **Chronic kidney disease** | 0 (0.0) |
| **Smoke** | 5 (2.9) |
| **Alcohol** | 6 (3.5) |
| **Dementia** | 0 (0.0) |
| **Previous stroke/TIA** | 0 (0.0) |
| **Prestroke mRs** | 0 (0.0) |
| **Antiplatelet drugs** | 0 (0.0) |
| **Anticoagulant drugs** | 0 (0.0) |
| **Statins** | 0 (0.0) |
| **Systolic pressure** | 7 (4.1) |
| **Diastolic pressure** | 7 (4.1) |
| **Creatinine** | 2 (1.2) |
| **Hemoglobin** | 0 (0.0) |
| **Leucocytes** | 0 (0.0) |
| **Neutrophils** | 0 (0.0) |
| **Monocytes** | 0 (0.0) |
| **Lymphocytes** | 0 (0.0) |
| **Platelets** | 0 (0.0) |
| **INR** | 3 (1.8) |
| **Admission glycemia** | 0 (0.0) |
| **Glycated hemoglobin** | 0 (0.0) |
| **Glycemic gap** | 0 (0.0) |
| **SHR** | 0 (0.0) |
| **GGHR** | 0 (0.0) |
| **Admission GCS** | 0 (0.0) |
| **Admission NIHSS** | 0 (0.0) |
| **Admission ICH volume** | 0 (0.0) |
| **ICH location** | 0 (0.0) |
| **ICH side** | 1 (0.6) |
| **Intraventricular hemorrhage** | 0 (0.0) |
| **Subarachnoid hemorrhage** | 0 (0.0) |
| **ICH score** | 0 (0.0) |
| **Hematoma expansion** | 0 (0.0) |
| **Early neurologic deterioration** | 0 (0.0) |
