# Supplementary material for: Stress hyperglycemia indexes and early neurological deterioration in spontaneous intracerebral hemorrhage
Source: Neurol Sci. 2025 Mar 19;46(7):3135–45. doi: 10.1007/s10072-025-08097-8 (PMC12152042; doi:10.1007/s10072-025-08097-8)
Supplement: Supplementary file 2 — Supplementary Material 2 [file 10072_2025_8097_MOESM2_ESM.docx]

| Glycemic gap | | | | | | |
| --- | --- | --- | --- | --- | --- | --- |
| **Model 1** | | | | | | |
|  | *Initial Model* |  |  | *Final Model* |  |  |
|  | OR | 95% CI | p-value | OR | 95% CI | p-value |
| Diastolic pressure | 0.96 | 0.936-1.001 | 0.07 | 0.97 | 0.934-0.998 | 0.05 |
| ICH score | 1.32 | 0.280-1.345 | 0.18 | ---- | ---- | ---- |
| GGAP ≥ 35.68 | 3.09 | 1.126-8.381 | 0.03 | 3.49 | 1.298-9.298 | 0.01 |
|  |  |  |  |  |  |  |
| **Model 2** | | | | | | |
|  | *Initial Model* |  |  | *Final Model* |  |  |
|  | OR | 95% CI | p-value | OR | 95% CI | p-value |
| Diastolic pressure | 0.97 | 0.938-1.004 | 0.10 | 0.97 | 0.934-0.998 | 0.05 |
| ICH score | 1.25 | 0.801-1.913 | 0.31 | ---- | ---- | ---- |
| GGAP ≥ 35.68 | 3.20 | 1.147-8.838 | 0.02 | 3.49 | 1.298-9.298 | 0.01 |
| Hematoma expansion | 1.92 | 0.693-5.349 | 0.20 | ---- | ---- | ---- |
|  |  |  |  |  |  |  |
| Stress hyperglycemia ratio | | | | | | |
| **Model 1** | | | | | |  |
|  | *Initial Model* |  |  | *Final Model* |  |  |
|  | OR | 95% CI | p-value | OR | 95% CI | p-value |
| Diastolic pressure | 0.97 | 0.933-1.000 | 0.06 | 0.96 | 0.930-0.996 | 0.04 |
| ICH score | 1.32 | 0.861-2.006 | 0.19 | ---- | ---- | ---- |
| SHR ≥ 1.15 | 2.95 | 1.117-8.159 | 0.03 | 3.32 | 1.286-9.058 | 0.01 |
|  |  |  |  |  |  |  |
| **Model 2** | | | | | | |
|  | *Initial Model* |  |  | *Final Model* |  |  |
|  | OR | 95% CI | p-value | OR | 95% CI | p-value |
| Diastolic pressure | 0.97 | 0.936-1.004 | 0.10 | 0.96 | 0.930-0.996 | 0.04 |
| ICH score | 1.25 | 0.801-1.913 | 0.31 | ---- | ---- | ---- |
| SHR ≥ 1.15 | 3.08 | 1.155-8.625 | 0.03 | 3.32 | 1.286-9.058 | 0.01 |
| Hematoma expansion | 1.98 | 1.017-1.325 | 0.19 | ---- | ---- | ---- |
|  |  |  |  |  |  |  |
| Glucose-glycated hemoglobin ratio | | | | | | |
| **Model 1** | | | | | | |
|  | *Initial Model* |  |  | *Final Model* |  |  |
|  | OR | 95% CI | p-value | OR | 95% CI | p-value |
| Diastolic pressure | 1.03 | 0.050-21.858 | 0.07 | 0.97 | 0.933-34.404 | 0.04 |
| ICH score | 1.34 | 0.877-2.018 | 0.17 | ---- | ---- | ---- |
| GGR ≥ 26.67 | 3.03 | 1.106-8.171 | 0.03 | 3.39 | 1.265-9.015 | 0.01 |
|  |  |  |  |  |  |  |
| **Model 2** | | | | | | |
|  | *Initial Model* |  |  | *Final Model* |  |  |
|  | OR | 95% CI | p-value | OR | 95% CI | p-value |
| Diastolic pressure | 0.97 | 0.938-1.004 | 0.10 | 0.97 | 0.933-34.404 | 0.04 |
| ICH score | 1.25 | 0.806-1.920 | 0.30 | ---- | ---- | ---- |
| GGR ≥ 26.67 | 3.13 | 1.128-8.624 | 0.03 | 3.39 | 1.265-9.015 | 0.01 |
| Hematoma expansion | 1.93 | 0.694-5.364 | 0.20 | ---- | ---- | ---- |
| OR: Odd Ratio; 95% CI: 95% confidence interval; ICH: intracerebral hemorrhage; GGAP: glycemic gap; SHR: stress hyperglycemia ratio; GGR: glucose-glycated hemoglobin ratio | | | | | | |

**Table S2 Multivariate analyses of early neurological deterioration (END) including stress hyperglycemia indexes as categoric variables**
